# Blocking GSDMD processing in innate immune cells but not in hepatocytes protects hepatic ischemia–reperfusion injury

**DOI:** 10.1038/s41419-020-2437-9

**Published:** 2020-04-17

**Authors:** Jichang Li, Jie Zhao, Min Xu, Meng Li, Bingrui Wang, Xiaoye Qu, Chang Yu, Hualian Hang, Qiang Xia, Hailong Wu, Xuehua Sun, Jinyang Gu, Xiaoni Kong

**Affiliations:** 1 Department of Liver Surgery, Renji Hospital, School of Medicine, Shanghai Jiao Tong University, Shanghai, China; 2 Central Laboratory, Department of Liver Diseases, Institute of Clinical Immunology, ShuGuang Hospital Affiliated to Shanghai University of Chinese Traditional Medicine, Shanghai, China; 3 Shanghai Key Laboratory for Molecular Imaging, Collaborative Research Center, Shanghai University of Medicine and Health Science, Shanghai, China; 4 Department of Transplantation, Xinhua Hospital, School of Medicine, Shanghai Jiao Tong University, Shanghai, China

**Keywords:** Diseases, Acute inflammation

## Abstract

Pyroptosis, a proinflammatory form of programmed cell death, plays important roles in the pathogenesis of many diseases. Inflammasome activation, which has been shown in hepatic ischemia–reperfusion injury (IRI), is demonstrated to be closely associated with pyroptosis, indicating that pyroptosis may occur and perform functions in hepatic IRI. However, there is no direct evidence showing the function of pyroptosis in hepatic IRI. In this study, by detecting the pyroptosis markers, we showed that pyroptosis may be induced during hepatic IRI. Furthermore, by adopting caspase-1 inhibitors, we showed that inhibition of pyroptosis could significantly ameliorate liver injury and suppress inflammatory response during hepatic IRI. Interestingly, caspase-1 inhibitors have no protective effects on in vitro hepatocytes under hypoxic reoxygenation condition. To investigate pyroptosis induced in which specific cell types may affect hepatic IRI, we generated hepatocyte-specific Gsdmd-knockout (Hep-Gsdmd^−/−^) and myeloid-specific Gsdmd-knockout (LysmCre^+^*Gsdmd*^f/f^) mice. Functional experiments showed that compared to control mice (Gsdmd^f/f^), there were alleviated liver injury and inflammation in LysmCre^+^*Gsdmd*^f/f^ mice, but not in AlbCre^+^*Gsdmd*^f/f^ mice. In parallel in vitro studies, cytokine expression and production decreased in bone-marrow-derived macrophages and Kupffer cells from LysmCre^+^*Gsdmd*^f/f^ mice compared to their controls. Our findings demonstrated that pyroptosis in innate immune cells aggravates hepatic IRI and implied that hepatic IRI could be protected by blocking pyroptosis, which may become a potential therapeutic target in the clinic.

## Introduction

Hepatic Ischemia–reperfusion injury (IRI) is still a major problem affecting the short- and long-term survival of patients who undergo liver transplantation and partial hepatectomy^1^. It has been proposed that there are two distinct phases of liver injury after warm I/R and a sterile inflammatory response^2^. The early phase of liver injury is characterized by ATP exhaustion, mitochondrial dysfunction and reactive oxygen species production, which occur in hepatocytes and directly lead to hepatocyte death^3^. The second phase of I/R injury is characterized by the activation of innate immune cells, including Kupffer cells and neutrophils, by released damage-associated molecular patterns (DAMPs) from injured or dead hepatocytes, resulting in elevated proinflammatory cytokine production and aggravated liver damage^4^.

Apoptosis and necrosis are two main modes of hepatocyte death following hepatic I/R injury^5^. Recently, pyroptosis was identified as a novel programmed cell death, which is mediated by the activation of inflammasome^6^. Earlier in 2001, Brennan and Cookson^7^ first used “pyroptosis” to name this cell death pattern^7^. When cells undergo pyroptosis, cells become swollen and activated caspase-1 cleaves full-length gasdermin *D* (GSDMD) to GSDMD-N terminus, which in turn oligomerizes and assembles into pores on the plasma membrane, resulting in the release of a large amount of cell contents and the induction of inflammatory response^8,9^. On the other hand, during pyroptosis, the intracellular precursors of interleukin-1β (IL-1β) and IL-18 can also be cleaved by activated caspase-1 to form mature IL-1β and IL-18, which are released from the GSDMD pores on the plasma membranes and recruit immunocytes to further aggravate inflammatory response^10^. Signaling pathways activated in the pyroptosis process have been divided into the classical signaling pathway mediated by caspase-1 and the non-canonical signaling pathway mediated by caspase-4, -5, and -11^11–13^. Activation of inflammasome involves both canonical and non-canonical signaling pathways^12,14^. Although there is no direct evidence showing the presence and the effects of pyroptosis in hepatic IRI, inflammasome activation has been frequently reported in hepatic IRI, suggesting that pyroptosis might occur and play important roles in hepatic IRI^15^.

Gsdmd belongs to the gasdermin (GSDM) family and is activated and cleaved by inflammasome-associated inflammatory caspases^14^. The oligomerization of N-terminal domain of cleaved Gsdmd and subsequent drilling pores on the plasma membrane are the critical steps for the onset of cell pyroptosis^16^. Although pyroptosis was first discovered in macrophages, Gsdmd is ubiquitously expressed in different tissues and cells^13^. Therefore, pyroptosis may also occur in non-immune cells. Thus, in order to study the effect of pyroptosis in hepatocytes and innate immune cells, we generated Gsdmd-flox mice (Gsdmd^f/fl^) and crossed them with AlbCre^+^ or LysmCre^+^ mice to establish knockout mice with specific GSDMD depletion in hepatocytes or innate immune, respectively.

In this study, we investigated the role of pyroptosis in hepatic IRI. We demonstrated that pyroptosis inhibitors could significantly ameliorate liver injury and suppress inflammation response in hepatic IRI. By adopting hepatocyte-specific Gsdmd-knockout (AlbCre^+^*Gsdmd*^f/f^) and myeloid-specific Gsdmd-knockout (LysmCre^+^*Gsdmd*^f/f^) mice, we found that LysmCre^+^*Gsdmd*^f/f^ mice rather than AlbCre^+^*Gsdmd*^f/f^ mice exhibited an alleviated liver injury and inflammation in response to liver IRI. In line with these in vivo results, pyroptosis inhibitors have no protective effects on hepatocytes under hypoxic reoxygenation (H/R) injury condition, and cytokine production was decreased in bone-marrow-derived macrophages (BMDMs) from LysmCre^+^*Gsdmd*^f/f^ mice compared to their controls. To our knowledge, this is the first study to show that the caspase-1-GSDMD processing mainly occurs mainly in innate immune cells, but not in hepatocytes during hepatic IRI, and demonstrate that blockage of the caspase-1-GSDMD processing in innate immune cells could improve hepatic IRI.

## Result

### Caspase-1-GSDMD processing was induced in the mouse hepatic IRI model

**Fig. 1:**
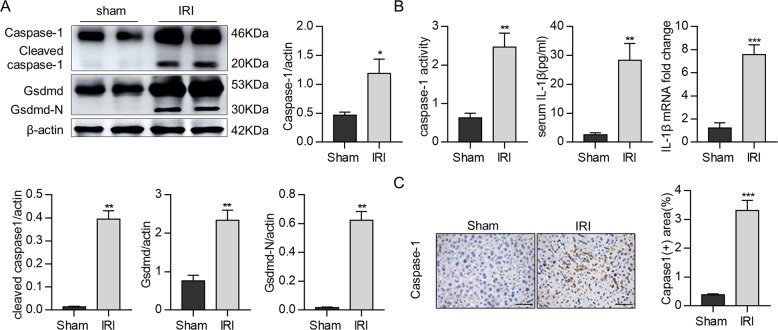
Caspase-1-GSDMD processing was induced in the mouse hepatic IRI model. C57/B6 mice were subjected to hepatic IRI model. **a** Gsdmd-full, Gsdmd-N, caspase-1, and cleaved caspase-1 expression in liver tissues were detected by Western blotting. **b** Serum levels of IL-1β, caspase-1 activity, and mRNA levels of IL-1β were measured in liver tissues. **c** Representative images of caspase-1 staining (scale bar, 30 μm), the area of caspase-1-positive cells in the livers were also determined. All data are shown as the mean ± SD, *n* (sham) = 4 mice per group, *n* (IRI) = 6 mice per group. ***P < 0.001, **P < 0.01 and *P < 0.05.

To investigate whether caspase-1-GSDMD processing is involved in hepatic IRI, we first examined the expression of caspase-1 and Gsdmd (markers of pyroptosis^17^) in the liver tissues in a hepatic IRI mouse model. As shown in Fig. 1a, IRI could induce the expression of both caspase-1 and full-length GSDMD and their processing. Time-course assays showed that the cleavage of caspase-1 and GSDMD, respectively, peaked at 4 and 6 h after reperfusion (Supplementary Fig. 4A). In addition, the cleavage of caspase-11 was not detectable during hepatic IRI (Supplementary Fig. 4B). Correspondingly, the serum levels of caspase-1 activity and mature IL-1β^18^ were significantly increased in the IRI group compared to the sham control (Fig. 1b). In addition, immunohistochemical staining also showed induction of caspase-1 in liver tissues in the hepatic IRI group compared to the sham group (Fig. 1c). Thus, our results show the possible induction of pyroptosis at least of caspase-1-GSDMD processing during hepatic IRI.

### Caspase-1 inhibitor protects mice from hepatic IRI

**Fig. 2:**
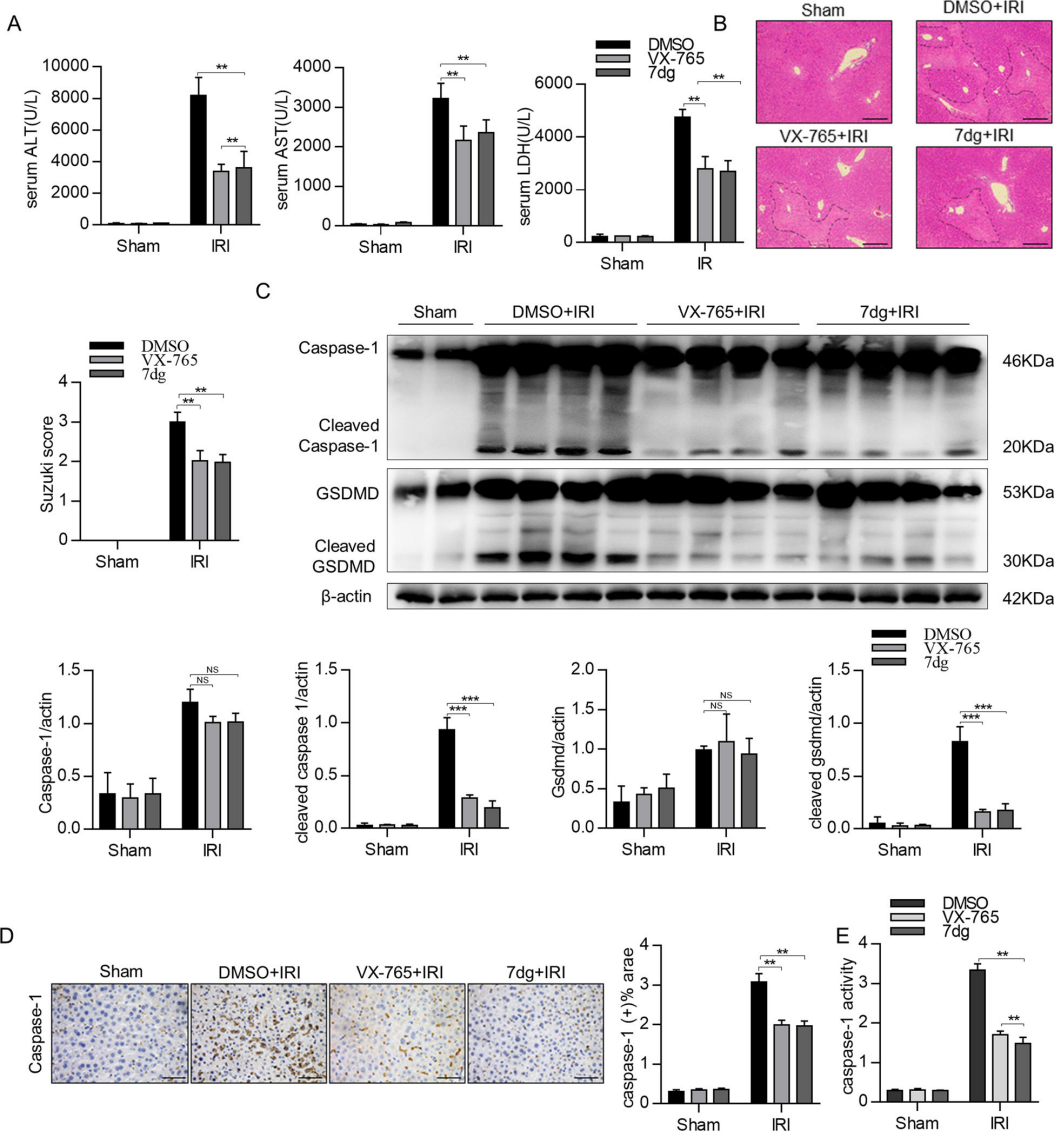
Caspase-1 inhibitor protects mice from hepatic IRI. **a** Hepatocellular function in serum samples was evaluated by detecting ALT, AST, and LDH levels. **b** Representative images of hematoxylin and eosin (HE)-stained liver tissues in different groups (scale bar, 100 μm), *n* = 4 mice per group. **c** Gsdmd-full, Gsdmd-N, and caspase-1 expression in liver tissues were detected by Western blot. **d** Representative image of caspase-1 staining was shown (scale bar, 30 μm). **e** Caspase-1 activities were measured in mice hepatic IRI liver tissues. All data are shown as the mean ± SD. ***P  < 0.001, **P < 0.01 and NS not significant.

Next, we sought to investigate the pathogenic role of caspase-1-GSDMD processing during hepatic IRI. Previous studies have shown that VX-765 is a small-molecule inhibitor that inhibits both expression and activity of caspase-1^19^, and 7dg is an inhibitor of caspase-1 activation by inhibiting PKR activity in a manner distinct from known PKR kinase inhibitors^20^. To investigate whether inhibition of caspase-1-GSDMD processing could improve hepatic IRI, we first performed dose–response assays of VX-765 and 7dg in the hepatic IRI mouse model. As shown in Supplementary Fig. 3, indicated doses of VX-765 or 7dg were administered 1 h before in the liver IRI model, and alanine aminotransferase/aspartate transaminase (ALT/AST) levels reflecting the severity of liver injury were measured. VX-765 (50 mg/kg) and 7dg (10 mg/kg) were chosen for in vivo treatment. VX-765 or 7dg pre-treatment could significantly improve liver injury induced by ischemia–reperfusion as evidenced by reduced serum ALT/AST levels (Fig. 2a), Suzuki’s histological scores (Fig. 2a) and TUNEL (terminal deoxynucleotidyl transferase dUTP nick-end labeling) staining signals (Supplementary Fig. 2A) in the treated groups compared to sham controls. Meanwhile, we also determined whether VX-765 or 7dg pre-treatment could affect the processing of caspase-1 and GSDMD during hepatic IRI (Supplementary Fig. 3E, F). Western blotting assays showed that VX-765 or 7dg pre-treatment significantly inhibited processing of caspase-1 and GSDMD in liver tissues (Fig. 2c). In line with this result, immunohistochemical staining in liver sections also showed decreased caspase-1 processing post VX-765 or 7dg pre-treatment (Fig. 2d). Meanwhile, decreased caspase-1 activity was also observed after VX-765 or 7dg treatment (Fig. 2e). The results suggest that the pre-treatment of caspase-1 inhibitors could protect hepatic IRI possibly by inhibiting the caspase-1-GSDMD processing.

### Caspase-1 inhibitors alleviate inflammatory responses in mice hepatic IRI

**Fig. 3:**
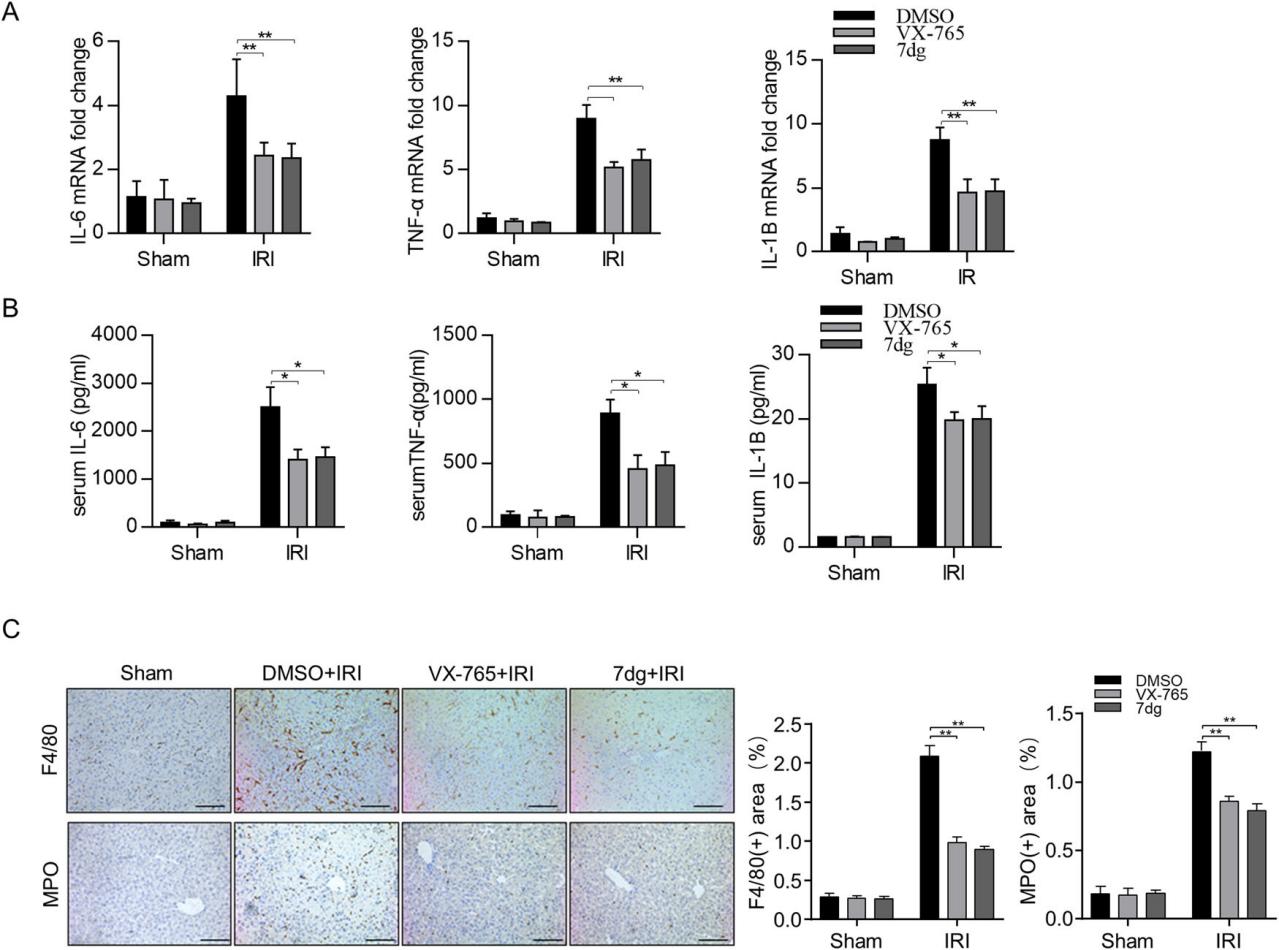
Caspase-1 inhibitors alleviate inflammatory responses in mice hepatic IRI. **a** mRNA levels of TNF-α, IL-1β, and IL-6 were determined by quantitative real-time PCR. **b** Indicated serum cytokines’ levels were measured by ELISA, *n* = 6 mice per group. **c** Representative images of F4/80 and MPO staining in liver tissue sections (scale bar, 50 μm), positive cells of MPO and F4/80 were determined, *n* = 4 mice per group. All data are shown as the mean ± SD. **P < 0.01 and *P < 0.05.

Given that inflammatory response plays an important role in exacerbating hepatic IRI, we further analyzed the levels of proinflammatory cytokines, such as IL-1β, IL-6, and tumor necrosis factor-α (TNF-α). Quantitative real-time (QPCR) and enzyme-linked immunosorbent assay (ELISA) assays showed that the induction of IL-1β, IL-6 and TNF-α was significantly reduced after VX-765 and 7dg treatment compared to vehicle controls (Fig. 3a, b). Next, we investigated the hepatic infiltration of immune cells by immunohistochemical staining of myeloperoxidase (MPO) (a marker of neutrophils) and F4/80 (a marker of macrophages). As shown in Fig. 3c, hepatic infiltration of neutrophils and macrophages was also significantly reduced in groups treated with caspase-1 inhibitors after hepatic IRI. Therefore, our results demonstrate that inhibitors of caspase-1 could alleviate the inflammatory responses of liver IRI.

### Caspase-1 inhibitors have no protective effects on hepatocytes in H/R treatment

**Fig. 4:**
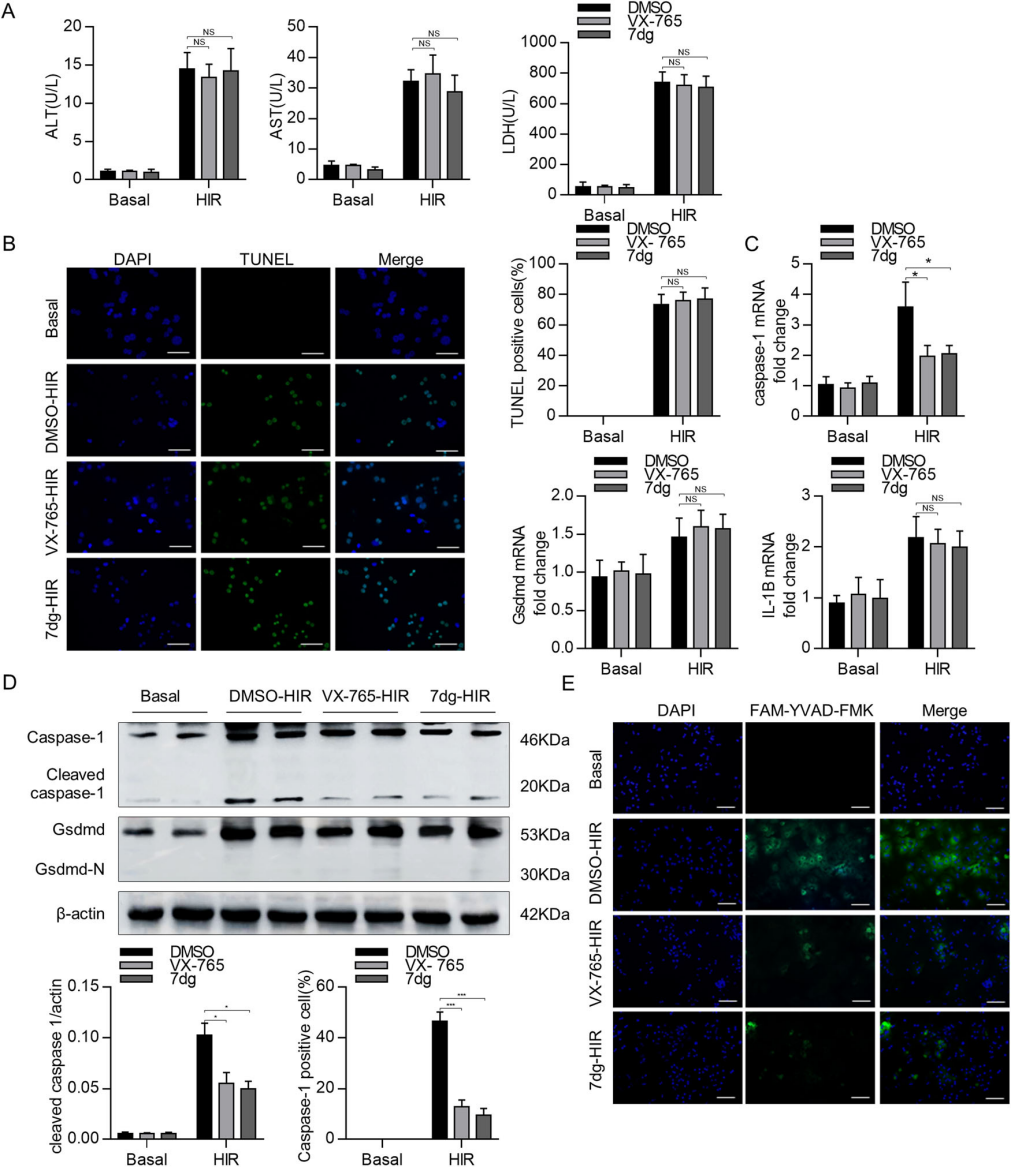
Caspase-1 inhibitors have no protective effects on hepatocytes in hypoxic reoxygenation (H/R) treatment. Primary hepatocytes were subjected to H/R injury and in the presence or absence of VX-765 or 7dg. **a** Supernatant ALT, AST, and LDH levels were measured, *n* = 6 mice per group. **b** Representative fluorescence images of TUNEL staining in primary hepatocytes. TUNEL-positive hepatocytes were expressed as a percentage of the total hepatocytes (scale bar, 30 μm). **c** mRNA levels of caspase-1, Gsdmd, and IL-1β were determined by quantitative real-time PCR, *n* = 6 mice per group. **d** Caspase-1 expression was analyzed by Western blot. **e** Representative fluorescence images of caspase-1 staining in primary hepatocytes (scale bar, 30 μm), *n* = 3 mice per group. All data are shown as the mean ± SD. ***P < 0.001, *P < 0.05, and NS not significant.

As injury and death of hepatocytes are the initial detrimental events during hepatic IRI and associated with inflammatory response, we then assessed whether caspase-1 inhibitors could directly protect hepatocytes. In vitro H/R treatment was performed to mimic in vivo IRIs and hepatocyte injury was determined by measuring the levels of lactic acid dehydrogenase (LDH), ALT, and AST. As shown in Fig. 4a, VX-765 and 7dg treatment had no protective effect on H/R-induced hepatocyte injury. Meanwhile, TUNEL staining also showed that VX-765 and 7dg treatment could not improve DNA damage in hepatocytes induced by hypoxia reoxygenation (Fig. 4b). QPCR assays showed that although the expression of caspase-1, *Gsdmd*, and *IL-1β* in hepatocytes was increased after H/R treatment, VX-765 and 7dg only inhibited caspase-1 expression, but had no effect on *Gsdmd* and *IL-1β* expression (Fig. 4c). Meanwhile, western blotting assays showed that H/R treatment significantly increased the production of caspase-1 and full-length GSDMD, and the cleavage of caspase-1, but the processing of full-length GSDMD was undetectable in hepatocytes in response to H/R treatment (Fig. 4d). VX-765 and 7dg treatment inhibited the levels of caspase-1 and cleaved caspase-1, but had no effect on GSDMD expression and processing (Fig. 4d). Moreover, immunofluorescence assays showed increased caspase-1 activity in response to H/R, which was decreased in VX-765- and 7dg-treated hepatocytes (Fig. 4e). These results indicated that although caspase-1 expression and processing were significantly induced in hepatocytes during H/R treatment, its downstream GSDMD processing did not occur, suggesting that GSDGD processing may not occur in hepatocytes during liver IRI, and caspase-1 inhibitors have no protective effects on hepatocytes in response to H/R treatment.

### Hepatocyte-specific depletion of *Gsdmd* mice (AlbCre^+^*Gsdmd*^f/f^) does not protect hepatic IRI

**Fig. 5:**
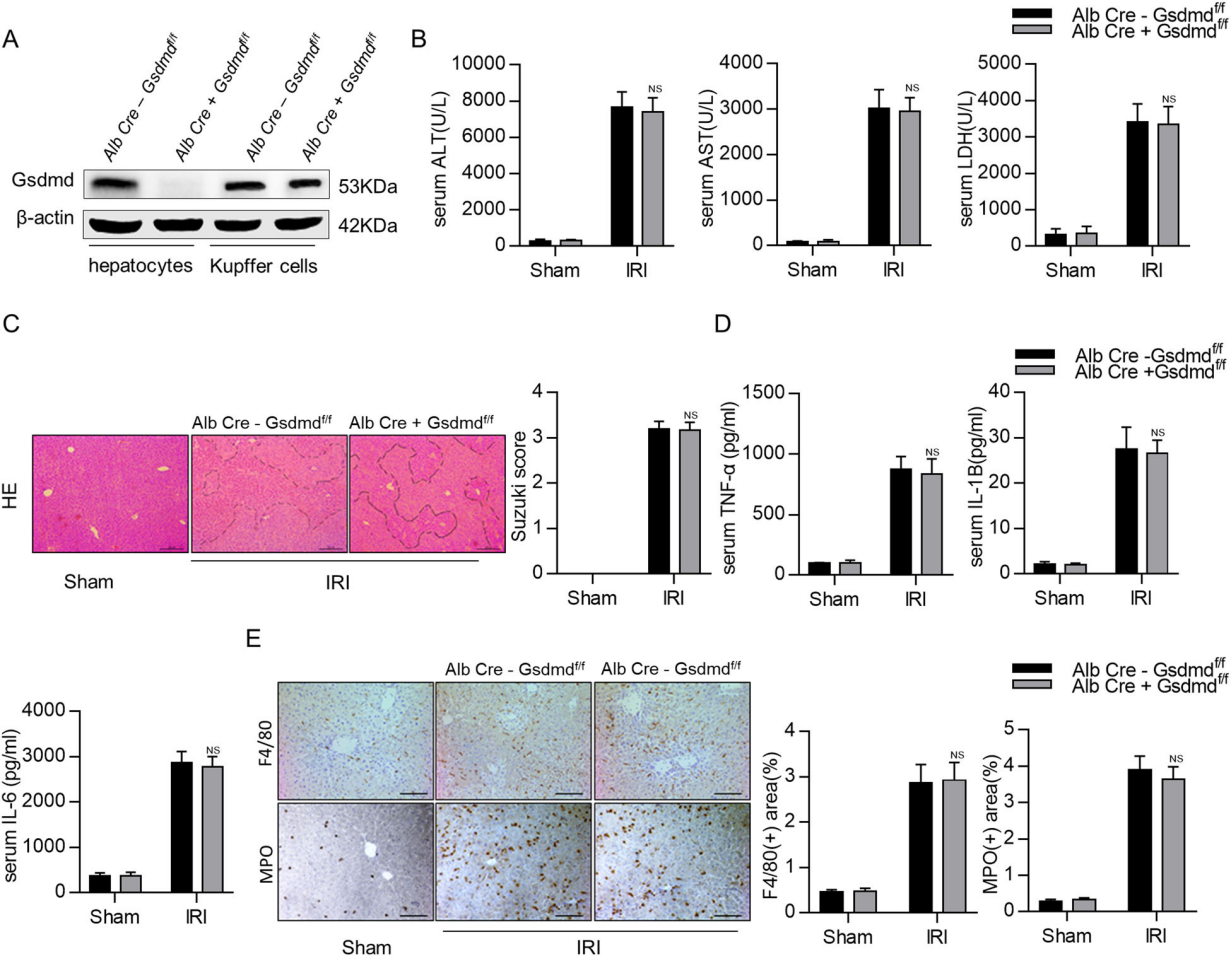
Hepatocyte-specific depletion of Gsdmd mice (AlbCre^+^*Gsdmd*^f/f)^ does not protect hepatic IRI. **a** Gsdmd expression in hepatocytes and liver Kupffer cells were measured by Western blot. **b** Serum levels of ALT, AST, and LDH were measured. **c** Representative images of hematoxylin and eosin (HE) staining (scale bar, 100 μm). **d** Serum cytokine levels were measured by ELISA. **e** Representative images of F4/80 and MPO staining in liver tissues (scale bar, 50 μm); MPO- and F4/80-positive cells were determined. All data are shown as the mean ± SD, *n* = 5 mice per group. NS not significant.

To investigate whether blocking the caspase-1-GSDMD processing in hepatocytes improves hepatic IRI, we generated hepatocyte-specific *Gsdmd*-knockout (AlbCre^+^*Gsdmd*^f/f^) mice by crossing *Gsdmd*^f/f^ mice with AlbCre^+^ mice. As shown in Fig. 5a, hepatocyte-specific GSDMD depletion was confirmed by western blotting. Compared to AlbCre^−^*Gsdmd*^f/f^ control mice, AlbCre^+^*Gsdmd*^f/f^ mice showed comparable liver injuries in response to hepatic IRI as evidenced by the serum levels of ALT, AST, and LDH (Fig. 5b). Consistently, there were no significant differences in liver necrosis areas and Suzuki scores between AlbCre^−^*Gsdmd*^f/f^ control mice and AlbCre^+^*Gsdmd*^f/f^ mice (Fig. 5c and Supplementary Fig. 2B). Meanwhile, similar serum levels of inflammatory factors, such as IL-6, TNF-α, and IL-1β, were observed between AlbCre^−^*Gsdmd*^f/f^ and AlbCre^+^*Gsdmd*^f/f^ mice (Fig. 5d). AlbCre^−^*Gsdmd*^f/f^ and AlbCre^+^*Gsdmd*^f/f^ mice showed comparable levels of cleaved caspase-1 and GSDMD (Supplementary Fig. 4c). Immunohistochemical staining showed that there were no differences in hepatic infiltrated immune cells in both genotypes (Fig. 5e). All these results clearly indicate that GSDMD depletion in hepatocytes does not alleviate hepatic IRI.

### *Gsdmd* deficiency in myeloid cells (LysmCre^+^*Gsdmd*^f/f^) improves hepatic IRI

**Fig. 6:**
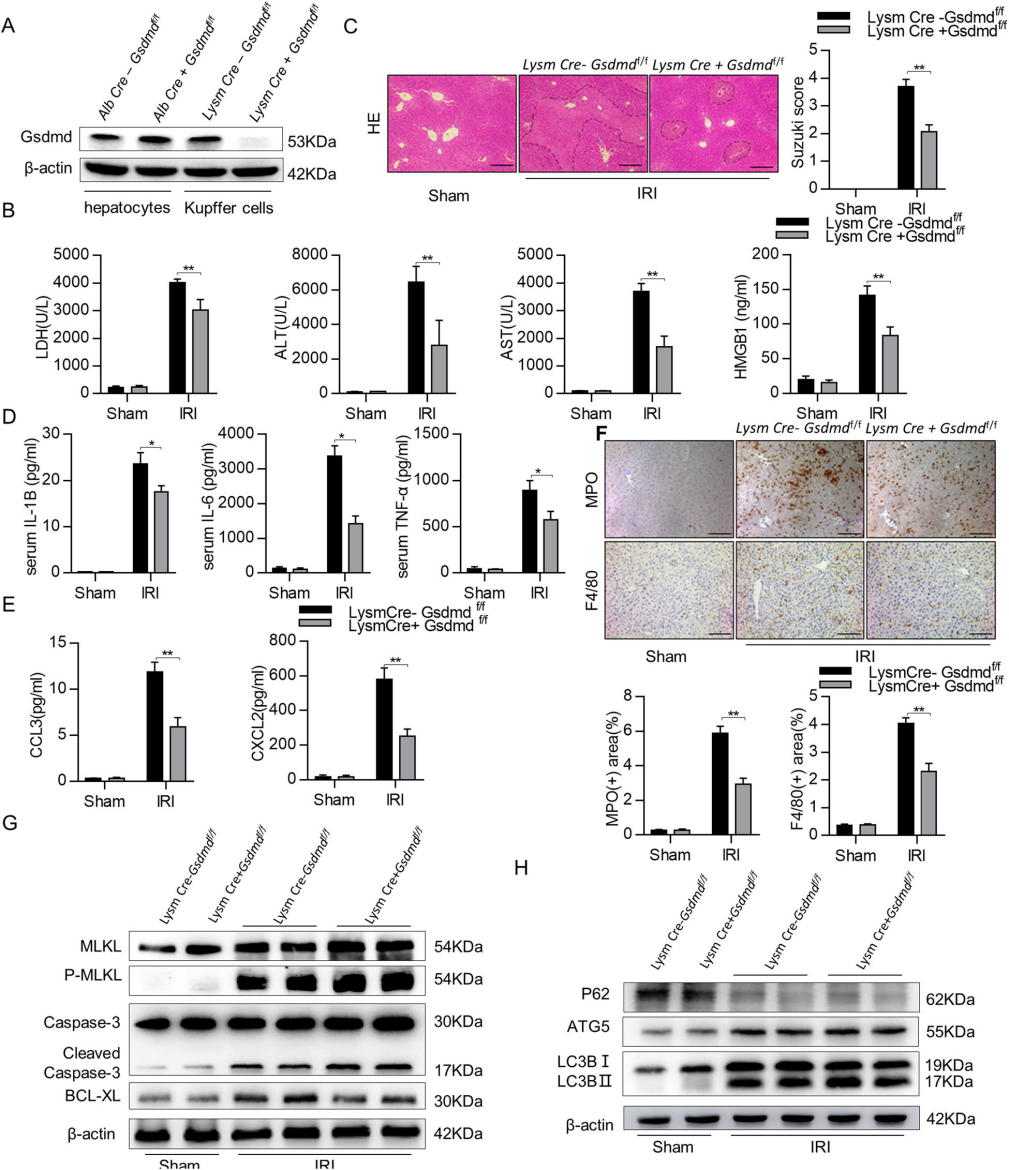
Gsdmd deficiency in myeloid cells (LysmCre^+^*Gsdmd*^f/f^) improves hepatic IRI. **a** Gsdmd expression in hepatocytes and liver Kupffer cells were measured by Western blot. **b** Serum levels of HMGB1, ALT, AST, and LDH. **c** Representative images of hematoxylin and eosin (HE) staining (scale bar, 100 μm). **d** Serum cytokine levels were measured by ELISA. **e** Serum chemokines levels were measured by ELISA. **f** Representative images of F4/80 and MPO staining liver tissues (scale bar, 50 μm); MPO- and F4/80-positive cells were determined. **g** MLKL, P-MLKL, caspase-3, and BCL-XL expression in the liver tissues were measured by Western blot. **h** Autophagy-related proteins P62, ATG5, and LC3 expression in the liver tissues were measured by Western blot. All data are shown as the mean ± SD, *n* = 6 mice per group. **P < 0.01 and *P < 0.05.

Having demonstrated that GSDMD depletion in hepatocytes does not protect hepatic IRI, we then investigated whether GSDMD depletion in innate immune cells could improve hepatic IRI. By crossing *Gsdmd*^f/f^ mice with LysmCre^+^ mice, we specifically knocked out *Gsdmd* in mouse innate immune cells. As shown in Fig. 6a, GSDMD depletion was specifically observed in Kupffer cells. As shown in Fig. 6b, compared to LysmCre^−^*Gsdmd*^f/f^ control mice, serum levels of ALT, AST, high-mobility group box-1 (HMGB1), and LDH, which reflect hepatic injury, were significantly decreased in LysmCre^+^*Gsdmd*^f/f^ mice. Liver necrosis areas and Suzuki’s histological scores were also remarkably decreased in LysmCre^+^*Gsdmd*^f/f^ mice (Fig. 6c and Supplementary Fig. 2C). Correspondingly, the serum levels of proinflammatory cytokines (TNF-α, IL-6, and IL-1β), chemokines (CCL3 and CXCL2), and hepatic infiltrated immune cells (macrophages and neutrophils) were significantly lower in LysmCre^+^*Gsdmd*^f/f^ mice compared to control ones (Fig. 6d–f). Meanwhile, although there was no difference in the levels of cleaved caspase-1, the levels of the cleaved GSDMD was significantly decreased in LysmCre^+^*Gsdmd*^f/f^ mice compared with LysmCre^−^Gsdmd^f/f^ mice (Supplementary Fig. 4C). In addition, we examined the levels of other cell death pathways, such as necroptosis and apoptosis, by detecting protein levels of P-MLKL, cleaved caspase-3, and Bcl-XL. As shown in Fig. 6g, there was a comparative increased in these apoptotic and necroptotic parameters in both LysmCre^−^*Gsdmd*^f/f^ and LysmCre^+^*Gsdmd*^f/f^ mice during hepatic IRI. Meanwhile, the induction of autophagy during hepatic IRI was similar in both genotypes (Fig. 6h). All these results clearly indicated that GSDMD depletion in innate immune cells plays protective role during hepatic IRI.

### *Gsdmd* deficiency inhibits cytokine production in macrophages

**Fig. 7:**
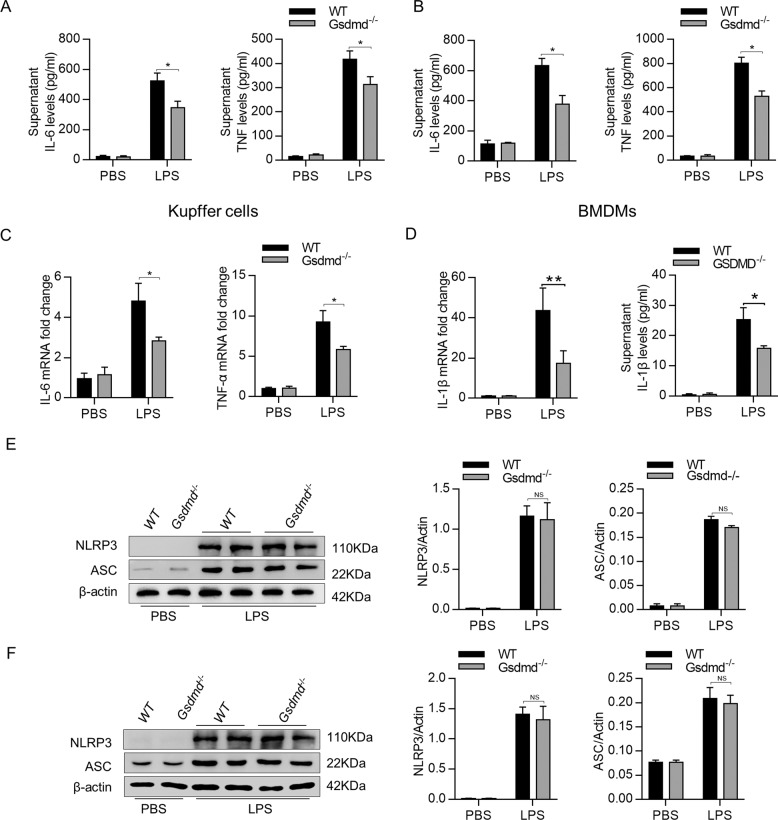
Gsdmd deficiency inhibits cytokine production in macrophages. **a**, **b** Same number of BMDMs and Kupffer cells were isolated from Gsdmd^f/f^ and LysmCre^+^*Gsdmd*^f/f^ mice and treated with LPS (100 ng/ml) for 6 h; levels of IL-6 and TNF-α in the supernatant were measured. **c** mRNA levels of TNF-α and IL-6 in BMDMs were determined by quantitative real-time PCR. **d** Expression and release of IL-1β were detected in macrophages treated with LPS for 6 h. **e**, **f** NLRP3 and ASC expression were measured by Western blot, *n* = 3 mice per group. **P < 0.01, *P < 0.05, and NS not significant.

To determine whether blocking caspase-1-GSDMD processing affects the immune response in innate immune cells, we treated mouse BMDMs and Kupffer cells with lipopolysaccharide (LPS) for 6 h to induce immune responses. As shown in Fig. 7a–c, BMDMs and Kupffer cells derived from Lysm Cre^+^*Gsdmd*^f/f^ mice produced much less IL-6 and TNF-α compared to them isolated from Lysm Cre^−^*Gsdmd*^f/f^ control mice. In addition, *Gsdmd* knockout significantly reduced the expression of pro-IL-1β and the release of mature IL-1β in BMDMs (Fig. 7d). It has been reported that the ASC/caspase-1 signaling pathway is associated with local inflammation during hepatic IRI^21,22^, so next we investigated whether this pathway is also involved in the decreased inflammation in BMDMs in LysmCre^+^*Gsdmd*^f/f^ mice. Western blotting assays showed that there were no differences in the expression of NLRP3 and ASC in BMDMs and Kupffer cells in both genotypes (Fig. 7d, e). These results suggest that GSDMD deficiency inhibits the inflammatory response in innate immune cells, which may contribute to the improved liver injury in LysmCre^+^*Gsdmd*^f/f^ mice during hepatic IRI.

## Discussion

The most important findings of this study are demonstrating: (1) the induction of caspase-1-GSDMD processing during hepatic IRI; (2) blocking caspase-1-GSDMD processing in innate immune cells, but not hepatocytes, could significantly ameliorate liver injury during hepatic IRI; (3) blocking caspase-1-GSDMD processing in innate immune cells could suppress inflammatory response in those cells. These findings suggest that caspase-1-GSDMD processing in innate immune cells plays a detrimental effect in hepatic IRI.

Hepatocyte damage and the subsequent inflammation are remarkable features in hepatic IRI. Pyroptosis has been implicated in various diseases, such as myocardial infarction, nonalcoholic fatty liver, and gastric cancer^23–25^, but whether pyroptosis plays a role in hepatic IRI remains unknown. In this study, we observed apparent caspase-1-GSDMD processing in liver tissues during hepatic IRI, suggesting the possible involvement of pyroptosis in hepatic IRI. Moreover, caspase-1 inhibitors showed a protective role in hepatic IRI. Pyroptosis can be induced by a non-canonical signaling pathway and a canonical signaling pathway^26,27^. The non-canonical signaling pathway is mediated by caspase-4/5 (in humans) or caspase-11 (in mice) and generally activated by bacterial signals^28,29^. The canonical signaling pathway is mediated by caspase-1 and activated by DAMPs derived from injured cells^23^. In this study, we found that the protein levels and the activity of caspase-1, but not caspase-11, were remarkably enhanced during hepatic IRI, suggesting the activation of the canonical pyroptosis signaling pathway.

Although pyroptosis was first discovered in immune cells^30^, *Gsdmd* is ubiquitously expressed in various cell types^31^, suggesting that non-immune cells may also undergo pyroptosis. Previous studies have shown that pyroptosis was induced in myocardial cells, adipocyte, and epithelial cells^17,32^. More recently, a study has reported that hepatocyte pyroptosis play important roles in alcoholic liver disease^33^. By adopting caspase-1 inhibitors, we demonstrated that blocking caspase-1-GSDMD processing improved hepatic IRI in vivo, but had no protective effects on H/R-induced hepatocyte injury in vitro, suggesting that caspase-1-GSDMD processing in hepatocytes may not be involved in hepatic IRI. By employing LysmCre^+^*Gsdmd*^f/f^e and AlbCre^+^*Gsdmd*^f/f^ mice, we clearly demonstrated that GSDMD depletion in innate immune cells, but not in hepatocytes, protects hepatic IRI (Supplementary Fig. 4E). This finding is consistent with a previous study showing that blocking inflammasome activation, an upstream key event of caspase-1 activation, in innate immune cells protects hepatic IRI^21^. We also found that GSDMD depletion in innate immune cells produced significantly less amount of inflammatory cytokines in response to LPS stimulation, which may account for the reduced inflammatory responses and liver injury in LysmCre^+^*Gsdmd*^f/f^ mice.

In addition to macrophages, neutrophils are also a type of crucial effector cells in the pathophysiology of liver IRI^34^. During IRI, Kupffer cells release proinflammatory cytokines and chemokines to recruit a large number of neutrophils to the liver injury sites^35^. The infiltrated neutrophils further release cytokines, including IL-1β and IL-18, to recruit more neutrophils, induce severe inflammatory responses, and subsequently amplify the tissue lesion^36^. Pyroptosis was first detected in macrophages, and then a number of studies have shown that pyroptosis may also occur in neutrophils, and was the main source of IL-1β secretion^37,38^. Our current study showed that in the liver ischemia–reperfusion model, the hepatic infiltration of neutrophils and the secretion of serum IL-1β were significantly reduced in LysmCre^+^*Gsdmd*^f/f^ mice. Gsdmd in neutrophils was also knocked out, suggesting that blocking pyroptosis in neutrophils may also reduce liver IRI.

Inflammasome is a multiprotein complex, which is activated by recognition of a variety of pathogen-associated molecular patterns (PAMPs) and DAMPs by nucleotide-binding oligomeric domain receptor-like receptors, resulting in the activation of caspase-1^39,40^. Therefore, inflammasome activation is an important process in mediating pyroptosis^41^. HMGB1 is an evolutionarily conserved and ubiquitously expressed DNA-binding protein. HMGB1 is known as one of the key endogenous DAMP molecules^42^ and may bind to a variety of TLRs, including TLR2, TLR4, and TLR9 for the initiation of an array of inflammatory responses^43,44^. HMGB1 release was also reported in ischemia-stressed cells^45^. Our study showed that in the mouse hepatic IRI model, the serum levels of HMGB1 in LysmCre^+^*Gsdmd*^f/f^ mice were significantly reduced, suggesting that liver injury and inflammation were reduced compared to LysmCre^−^*Gsdmd*^f/f^ mice. HMGB1 activates caspase-1 through TLR4^21^, and activated caspase-1 cleaves pro-IL-1β and pro-IL-18 to form mature IL-1β and IL-18, thereby expanding the inflammatory response^21^. Although there were no direct evidences of pyroptosis in hepatic IRI, the activation of inflammasome and production of IL-1β have been extensively reported^46,47^. Consistent with these previous studies, our study showed that caspase-1 and IL-1β levels were significantly increased after hepatic IR, suggesting that the activation of inflammasome and production of IL-1β in hepatic IRI-mediated effects were involved in pyroptosis.

In conclusion, our study showed that GSDMD-driven pyroptosis in innate immune cells plays an important role in the pathogenesis of hepatic IRI. These findings open up new insight into the treatment of hepatic IRI.

## Materials and methods

### Animals

Male WT C57BL/6 mice (8–10 weeks old) were purchased from Shanghai SLAC Co. Ltd (Shanghai, China). Gsdmd^f/f^ mice were generated and AlbCre^+^, LysmCre^+^ mice were purchased from Shanghai Biomodel Organism Science and Technology Development Co. Ltd. Strategy of Gsdmd^f/f^ mice was shown in Supplementary Fig. 1. All procedures involving animals were reviewed and approved by the Institutional Animal Care and Use Committee of Renji Hospital, School of medicine, Shanghai Jiao Tong University.

### Hepatic IRI mice model and treatment

The model of warm partial hepatic I/R injury was used as described in our previous study^48^. In brief, the control group (sham) only had free hepatic portal blood vessels after laparotomy, and did not block blood flow, the hepatic IRI group was freed from the hepatic portal vein and blocked the blood supply to the left lobe and mid-hepatic lobe for 90 min, and the blood vessels were then opened for 6 h. If the mice died before taking the sample, the sample was discarded. All operations were performed by the same operator, and the mice were fasted for 14 h before surgery. 7dg (10 mg/kg) (Sigma-Aldrich, St. Louis, MO, USA) or VX-765 (50 mg/kg) (MCE, NJ, USA) was administered intraperitoneally 1 h before liver ischemia. In vehicle group mice, a volume of 1% dimethyl sulfoxide (DMSO) equal to the volume of treatment was administered in the same manner.

### Biochemical measurement

Blood was collected by direct puncture of arteriae aorta and centrifuged at 12,000r for 5 min. Serum ALT, AST, and LDH levels were measured by microplate test kits (Nanjing Jiancheng Bioengineering Institute, Nanjing, China) according to the manufacturer’s instructions.

### Histology and immunohistochemical staining

Liver sections were stained with hematoxylin and eosin. The histological severity of liver injury was evaluated by the Suzuki’s criteria^37^. For immunohistochemistry analyses, liver sections were rehydrated and processed for an antigen-unmasking procedure, and then incubated with primary antibodies against MPO (Cell Signaling, Boston, MA, USA), cleaved caspase-1 (Abcam Cambridge, MA, USA), and F4/80 (AbD Serotec, Kidlington, UK) overnight at 4 °C, followed by horseradish peroxidase-conjugated secondary antibodies and observe immunoreactive cells with DAB (diaminobenzidine) under the microscope. For each stained section, three to six images from random fields were taken, and at least three mice per group were subjected to each experiment. Image-Pro Plus 6.0 was used for image analysis of sections.

### Hepatocyte and BMDM isolation, culture, and treatment

Primary hepatocyte isolation was performed as previously described^49^. The isolated cells were plated in dishes (2 × 10^6^ cells/6-cm dish), 6-well plates (5 × 10^5^ cells/well), or 24-well plates (5 × 10^4^ cells/well). To perform H/R in vitro, hepatocytes were cultured for 4 h at 37 °C in a modular incubator chamber (Biospherix, Lacona, NY, USA) gassed with 5% CO_2_ and 95% N_2_ for 4 h, and then hepatocytes were returned to the normoxic incubator for 6 h. To assess cells injury, LDH was measured by LDH Release Assay Kit (Beyotime, Shanghai, China) according to the manufacturer’s protocols. 7dg (5 μM) (Sigma-Aldrich, St. Louis, MO, USA) or VX-765 (50 μM) (MCE, NJ, USA) was administered 1 h before hepatocytes’ hypoxia; in vehicle-treated hepatocytes, a volume of 0.1% DMSO equal to the treatment was administered in the same manner. BMDMs were isolated as previously described^50^. BMDMs isolated from LysmCre^−^*Gsdmd*^f/f^ and LysmCre^+^*Gsdmd*^f/f^ were cultured in six-well plates at 3 × 10^6^ cells/well in 1640 medium supplemented with 10% fetal bovine serum. Then, they treated with 100 ng/ml LPS for 6 h, and the levels of IL-6 and TNF-α in the supernatant were detected.

### Quantitative RT-PCR

Total liver tissue RNA was extracted using TRIzol (Takara, Tokyo, Japan) reagent according to the manufacturer’s instructions. Complementary DNA (cDNA) was Synthesized using 500 ng of total RNA in the first-strand cDNA synthesis reaction with PrimeScript RT Teagent Kit (Takara, Tokyo, Japan). Reverse transcription-PCR (RT-PCR) was performed using the CFX 96 QPCR system (Bio-Rad, Hercules, CA, USA). A SYBR RT-PCR Kit (Takara, Tokyo, Japan) was used for QRT-PCR analysis. β-Actin was used as the standard for the relative expression levels for a target gene.

Primers used for RT-PCR analysis were as follows:

IL-1b forward: 5′-TGTAATGAAAGACGGCACACC

IL-1b reverse: 5′-TCTTCTTTGGGTATTGCTTGG

Tnf forward: 5′-TTCTATGGCCCAGACCCTCA

Tnf reverse: 5′-TTTGCTACGACGTGGGCTAC

IL-6 forward: 5′-GCTACCAAACTGGATATAATCAGGA

IL-6 reverse: 5′-CCAGGTAGCTATGGTACTCCAGAA

Caspase-1 forward: ACAAGGCACGGGACCTATG

Caspase-1 reverse: TCCCAGTCAGTCCTGGAAATG

Gsdmd forward: CCATCGGCCTTTGAGAAAGTG

Gsdmd reverse: ACACATGAATAACGGGGTTTCC

### Western blotting analysis

Western blotting analysis was performed as we previously described^51^. Primary antibodies were used against mouse caspase-1 (#ab179515), Gsdmd, Gsdmd-N (#ab209845) (Abcam Cambridge, MA, USA), NLRP3 (#15101), ASC (#67824) (Cell Signaling, Boston, MA, USA); for these specific proteins, β-actin (Sigma-Aldrich St. Louis, MO, USA) was used as a loading control.

### Enzyme-linked immunosorbent assay

ELISA Kits were used to detect mouse serum levels of IL-6, TNFα, and IL-1β (Neo Bioscience Technology, Shenzhen, China) according to the manufacturer’s protocols.

### Caspase-1 activity

Caspase-1 activity was detected in liver tissues. The activity was measured with Caspase-1 Assay Kit (Jiancheng Biotechnology, Nanjing, China) according to the manufacturer’s instructions

### Statistical analysis

The GraphPad Prism 6 software (GraphPad Software, San Diego, CA, USA) was used for statistical analysis. Results were presented as the means ± SEM, unless otherwise stated. Comparisons of two groups were performed using the unpaired Student’s *t* test, and the variance between the groups that are being statistically compared is similar. Survival curves were compared using the log-rank (Mantel–Cox) test. *P* values <0.05 were considered significant.

## Supplementary information

Strategy of the Gsdmd-knockout mice

Tunel staining of liver sections from hepatic ischemia-reperfusion injury

The dosing studies for caspasae-1 inhibitors used in vivo and vitro.

The expression of Pyroptosis related protein in hepatic tissue during ischemia-reperfusion.

LPS induces macrophage pyroptosis by activating the caspase-1-gsdmd pathway

Supplementary Figure Legends
