# Supplementary figures and images for: Blocking GSDMD processing in innate immune cells but not in hepatocytes protects hepatic ischemia–reperfusion injury
Source: Cell Death Dis. 2020 Apr 17;11(4):244. doi: 10.1038/s41419-020-2437-9 (PMC7165177; doi:10.1038/s41419-020-2437-9)

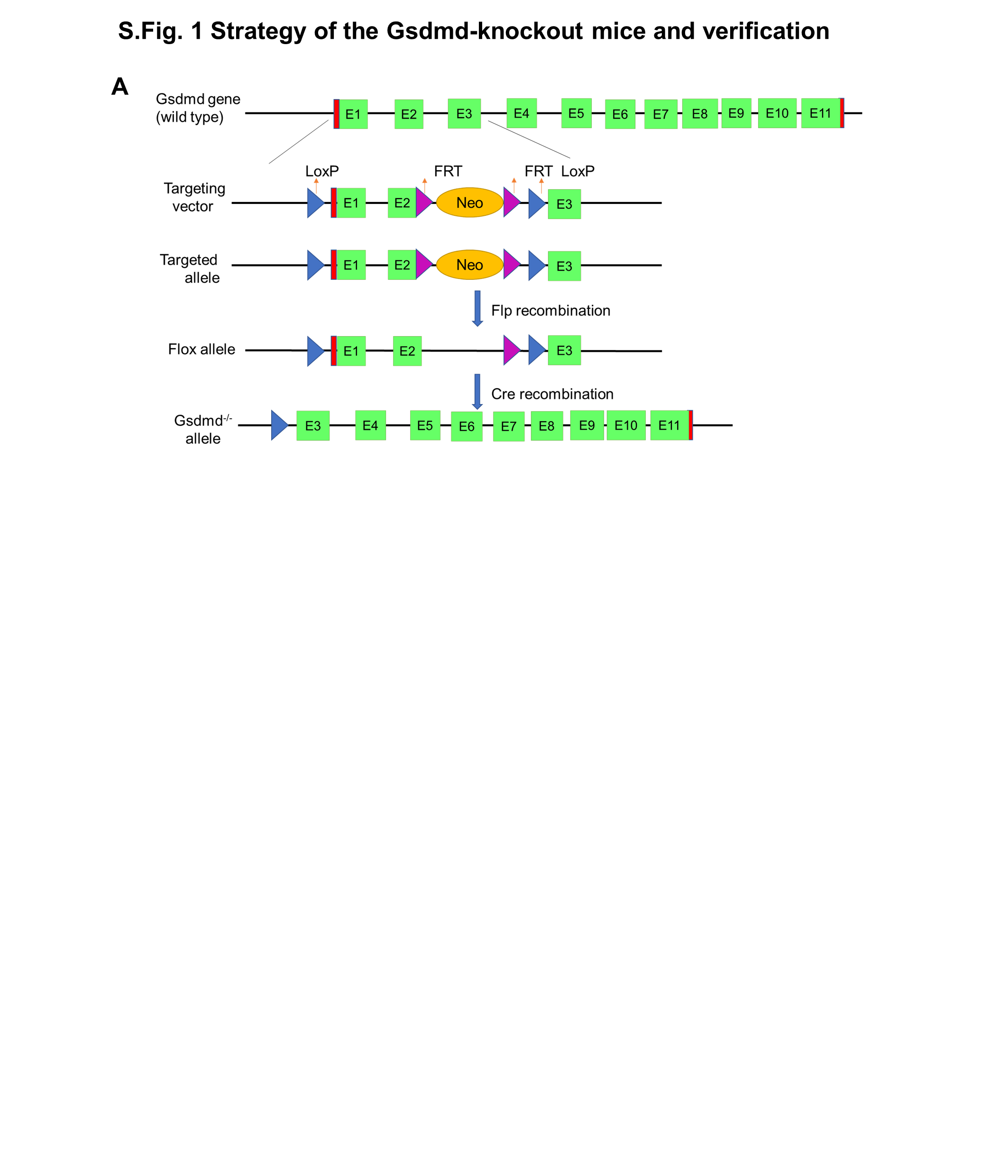

Supplement: Supplementary file 1 — Strategy of the Gsdmd-knockout mice [file 41419_2020_2437_MOESM1_ESM.tif]

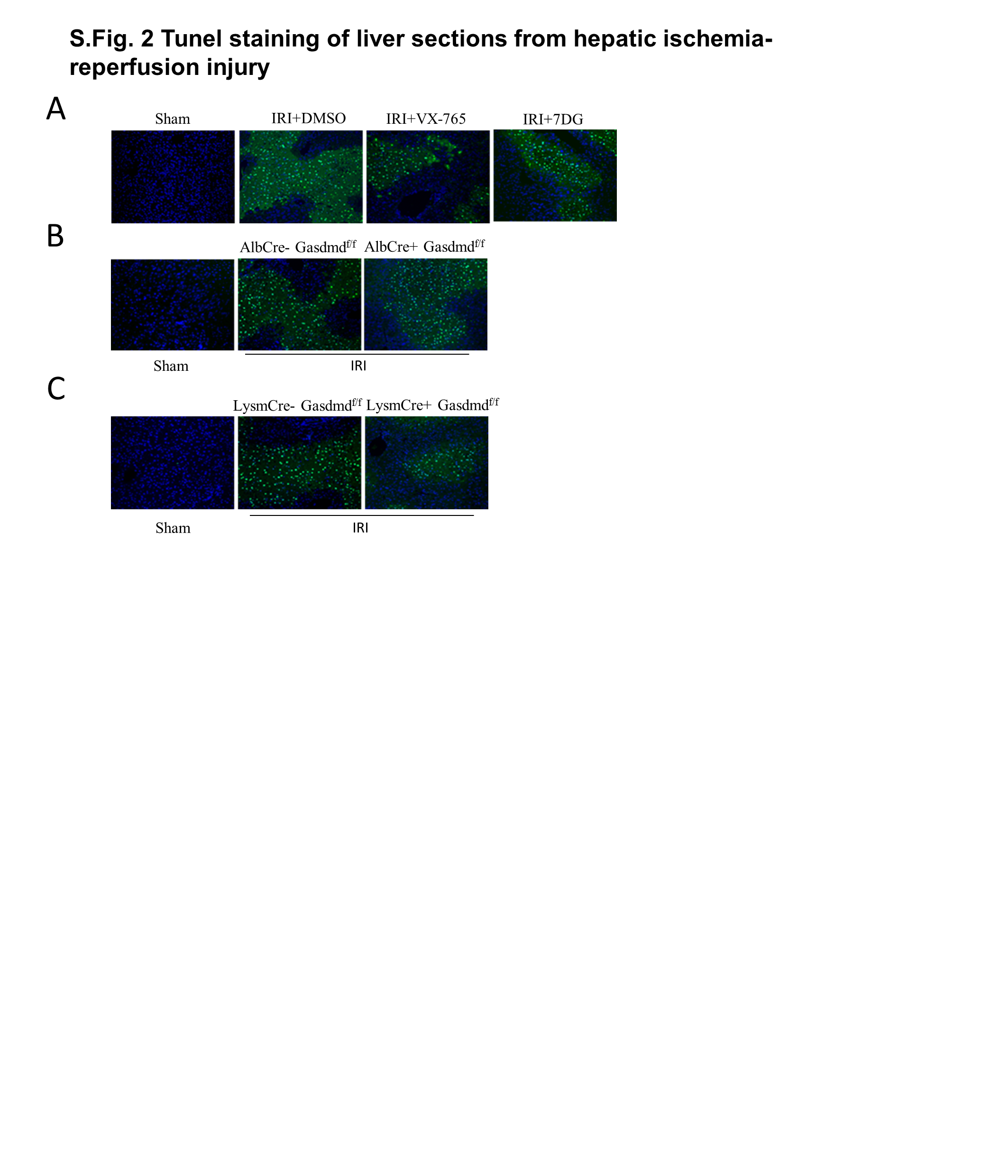

Supplement: Supplementary file 2 — Tunel staining of liver sections from hepatic ischemia-reperfusion injury [file 41419_2020_2437_MOESM2_ESM.tif]

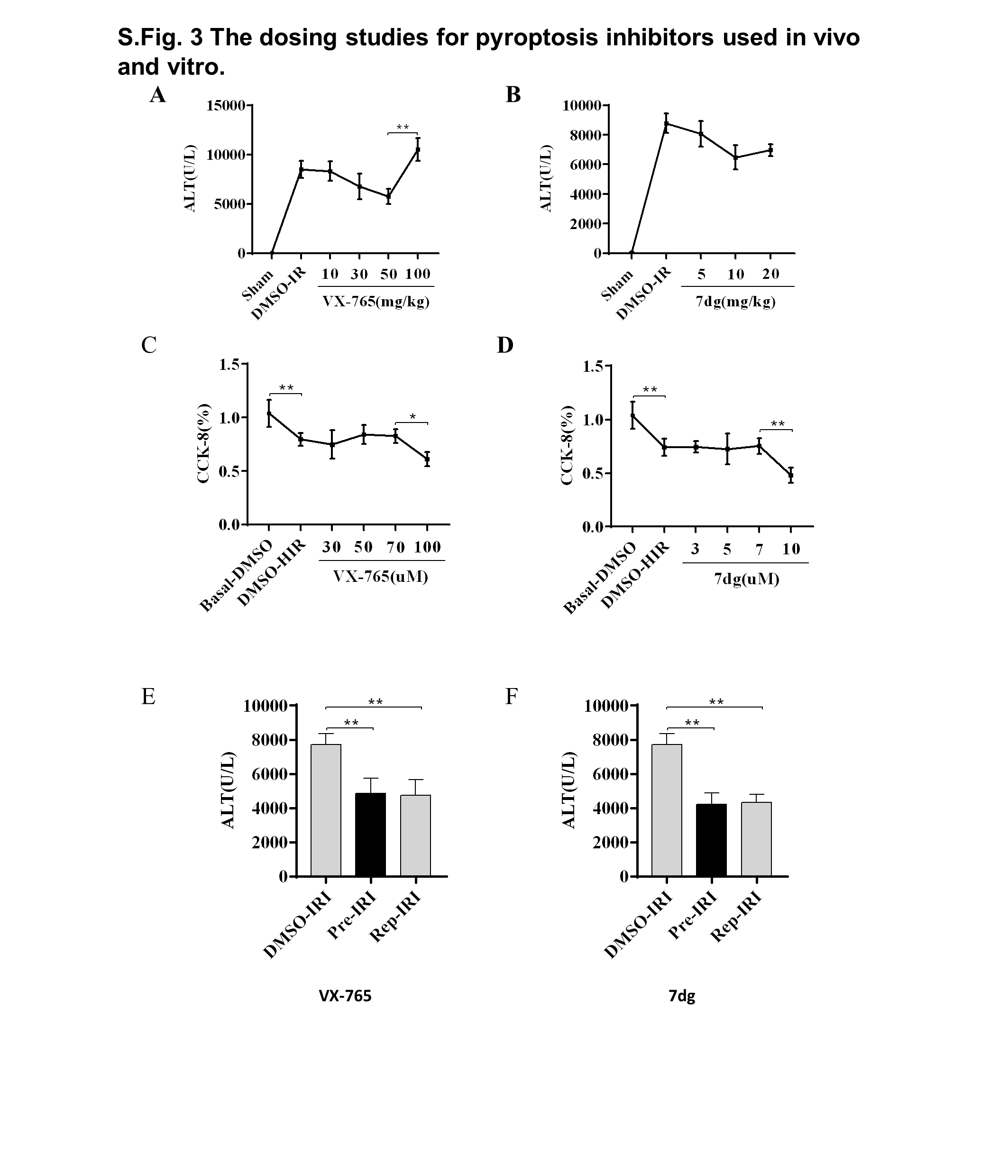

Supplement: Supplementary file 3 — The dosing studies for caspasae-1 inhibitors used in vivo and vitro. [file 41419_2020_2437_MOESM3_ESM.tif]

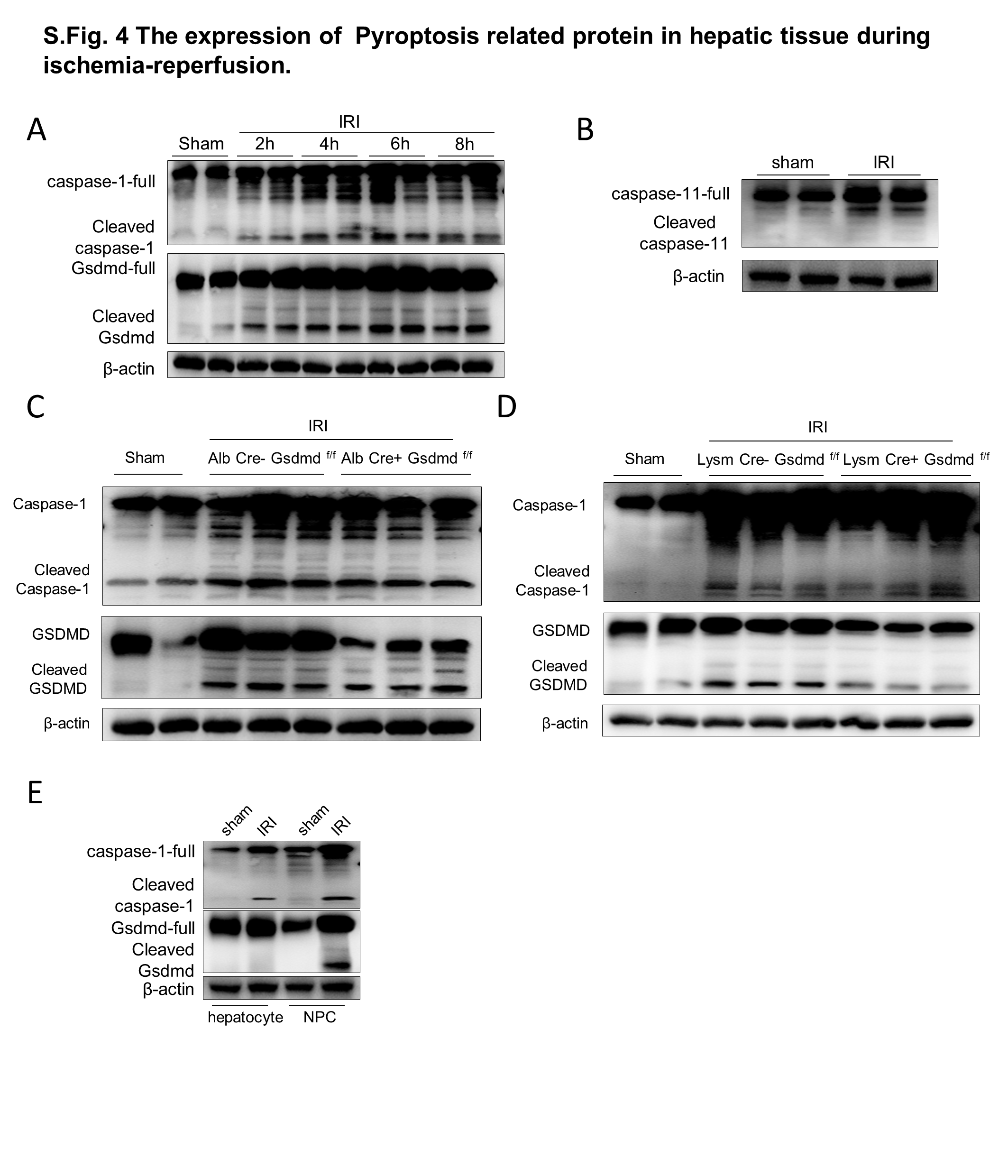

Supplement: Supplementary file 4 — The expression of Pyroptosis related protein in hepatic tissue during ischemia-reperfusion. [file 41419_2020_2437_MOESM4_ESM.tif]

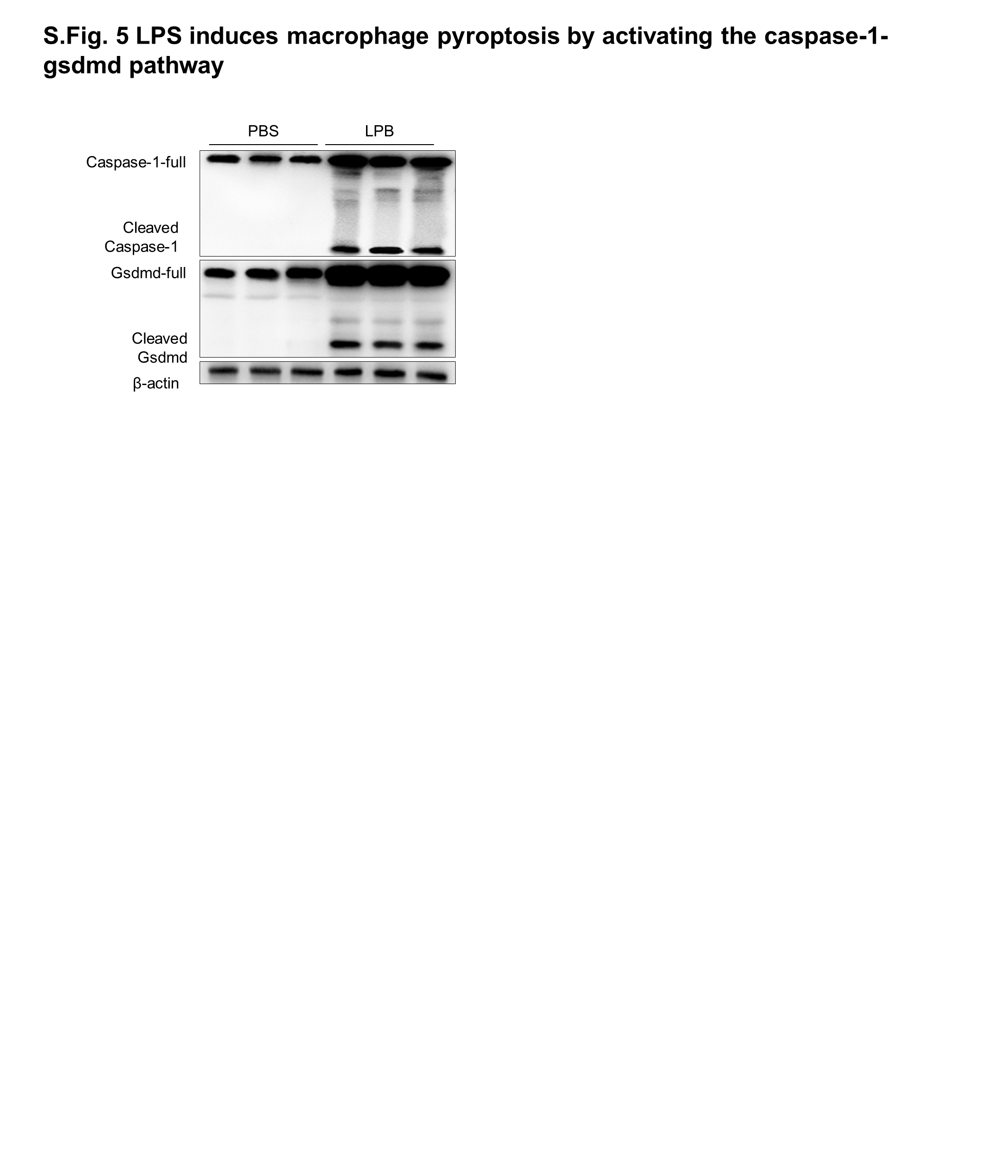

Supplement: Supplementary file 5 — LPS induces macrophage pyroptosis by activating the caspase-1-gsdmd pathway [file 41419_2020_2437_MOESM5_ESM.tif]
