# Supplementary material for: Blocking GSDMD processing in innate immune cells but not in hepatocytes protects hepatic ischemia–reperfusion injury
Source: Cell Death Dis. 2020 Apr 17;11(4):244. doi: 10.1038/s41419-020-2437-9 (PMC7165177; doi:10.1038/s41419-020-2437-9)
Supplement: Supplementary file 6 — Supplementary Figure Legends [file 41419_2020_2437_MOESM6_ESM.doc]

**S.Fig. 1 Strategy of the *Gsdmd*-knockout mice.** Strategy of the *Gsdmd*-knockout mice .

**S.Fig. 2 Tunel staining of liver sections from hepatic ischemia-reperfusion injury**

(A ) Tunel staining of liver sections from DMSO group and inhibitor group. (B) Tunel staining of liver sections from AlbCre- Gsdmd^f/f^ mice and AlbCre+ Gsdmd^f/f^ mice.(C) Tunel staining of liver sections from LysmCre- Gsdmd^f/f^ mice and LysmCre+ Gsdmd^f/f^ mice.

**S.Fig. 3 The dosing studies for caspasae-1 inhibitors used in vivo and vitro.** (A and B) Adopted different dosing inhibitors and injected intraperitoneally 1h before ischemia. Then mice were subjected to 90min of partial liver warm ischemia, followed by 6h of reperfusion. Serum levels of ALT were measured.(C and D) Pretreated them with different dosing VX-765 and 7dg or vehicle *in vitro*, followed by hypoxia reoxygenation (H/R) to mimic *in vivo* ischemia reperfusion injuries, cck8 was used to evaluate cell activity.(E and F) inhibitors are given at the time of reperfusion or pre-IRI, serum levels of ALT were measured.

**S.Fig. 4 The expression of Pyroptosis related protein in hepatic tissue during ischemia-reperfusion.**

(A ) Protein expression levels of cleaved caspase-1 and cleaved GSDMD in liver tissues of mice at different reperfusion times. (B) Caspase-11 and cleaved caspase-11 expression in liver tissues were detected by Western blotting. (C and D) Gsdmd-full, cleaved Gsdmd, caspase-1 and cleaved caspase-1 protein expression levels were detected in Gsdmd knockout mice at 6h after reperfusion.

**S.Fig. 5 LPS induces macrophage pyroptosis by activating the caspase-1-gsdmd pathway**

Gsdmd-full, Gsdmd-N, caspase-1 and cleaved caspase-1 expression in liver tissues were detected by Western blotting.
